# Supplementary material for: Proteomic and Transcriptomic Analyses to Decipher the Chitinolytic Response of Jeongeupia spp
Source: Mar Drugs. 2023 Aug 15;21(8):448. doi: 10.3390/md21080448 (PMC10455584; doi:10.3390/md21080448)
Supplement: Supplementary file 1 [file marinedrugs-21-00448-s001.zip › marinedrugs-2550935-supplementary.pdf]

Supplementary Materials

Table S1: Signal peptide prediction of the *J. wiesaeckerbachi* chitinolytic machinery with SignalP 6.0.**,** which recognizes conventional Sec, TAT and pilin-like translocons, processed with either of SPI, SPII or SPIII leader peptidases, respectively. GH = glycoside hydrolase family XX. LPMO = Lytic polysaccharide monooxygenase.

Gene ID	CAZy classification	Probability	Translocon/leader Peptidase	Cleavage site	Function
pgaptmp_000366	GH 18	96.8%	Sec/SPI	Pos. 23 - 24	Chitinase
pgaptmp_000371	GH 18	46.4%	Sec/SPI	Pos. 10 - 11	
pgaptmp_000372	GH 18	97.8%	Sec/SPI	Pos. 24 - 25	
pgaptmp_000389	GH 18	97.5%	Sec/SPI	Pos. 23 - 24	
pgaptmp_000635	GH 18	NA	NA	NA	
pgaptmp_000836	GH 18	97.2%	Sec/SPI	Pos. 21 -22	
pgaptmp_000837	GH 18	97.3%	Sec/SPI	Pos. 23 - 24	
pgaptmp_001504	GH 18	94.4%	Sec/SPI	Pos. 24 -25	
pgaptmp_001732	GH 18	94.9%	Sec/SPI	Pos. 34 -35	
pgaptmp_001841	GH 18	75.9%	Sec/SPI	Pos. 18 - 19	
pgaptmp_002137	GH 18	99.4%	Sec/SPII	Pos. 15 - 16	
pgaptmp_003083	GH 18	97.6%	Sec/SPI	Pos. 17 -18	
pgaptmp_001746	GH 18	NA	NA	NA	
pgaptmp_000302	GH 19	97.9%	Sec/SPI	Pos. 21 - 22	
pgaptmp_000680	GH 19	NA	NA	NA	
pgaptmp_001077	GH 19	97.6%	Sec/SPI	Pos. 20 - 21	
pgaptmp_000269	GH 20	NA	NA	NA	Hexo-saminidase
pgaptmp_000306	GH 20	NA	NA	NA	
pgaptmp_001731	GH 20	97.6%	Sec/SPI	Pos. 20 -21	
pgaptmp_000308	GH 3	NA	NA	NA	
pgaptmp_000872	GH 3	NA	NA	NA	
pgaptmp_001323	GH 3	NA	NA	NA	
pgaptmp_000148	LPMOAA10	98%	Sec/SPI	Pos. 22 - 23	LPMO

Table S2: Sequencing and mapping metrics per sample.

Sample	Carbon Source	Total HQ Reads	Mapped Reads	Unmapped Reads	Unique Reads
1	Colloidal Chitin	21.57M	20.11M (93.25%)	1.46M (6.75%)	19.69M (91.28%)
2	Colloidal Chitin	18.65M	17.80M (95.45%)	848.81K (4.55%)	17.37M (93.14%)
3	Glucose	21.70M	21.56M (99.33%)	144.42K (0.67%)	20.70M (95.37%)
4	Glucose	21.71M	21.55M (99.30%)	152.72K (0.70%)	20.67M (95.24%)

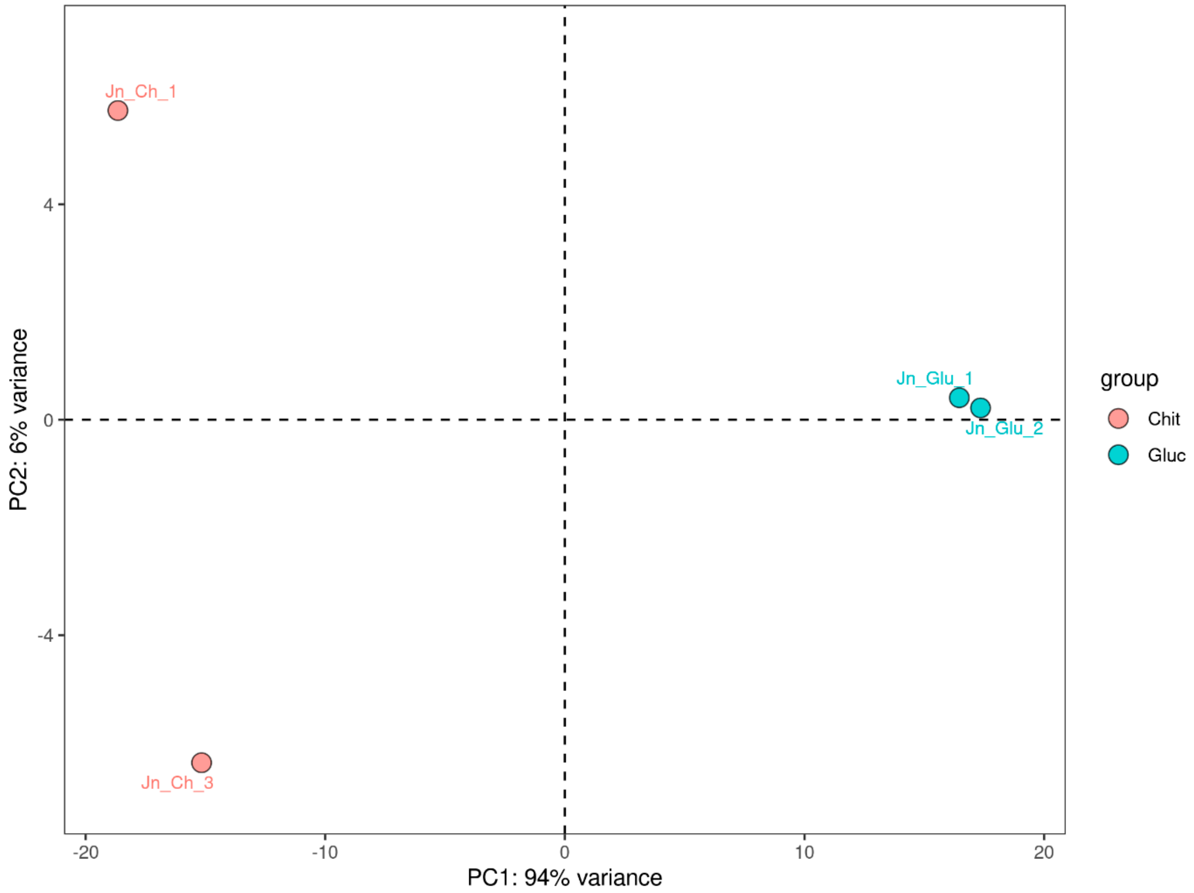

Figure S1: Principal Component Analysis (PCA) plot of the differential transcriptomics samples of *Jeongeupia wiesaeckerbachi*. Pink dots represent minimal colloidal chitin media derived transcriptomes, while light blue dots represent minimal glucose media derived transcriptomes. The chitin induced RNA sample 2 had to be omitted due to putative contamination. Therefore, a third sample "Jn-Ch_3" was sequenced and evaluated.

Table S3: Top 5 most variable transcripts of *Jeongeupia wiesaeckerbachi* in minimal media with glucose as exclusive carbon source. Transcripts were sorted according to the five lowest FDR corrected P-values. Additionally, all detected glucosamine metabolism related genes, which were upregulated under glucose media conditions, are listed in the lower half of the table.

**Carbon source**	**Rank**	**Adjusted *p*-value**	**log2 Fold Change**	**Gene ID**	**Description**
Glucose	**1**	1.96×10^{-47}	4.41	pgaptmp_000260	porin
	**2**	1.69×10^{-44}	4.31	pgaptmp_001037	hypothetical protein
	**3**	1.88×10^{-41}	5.61	pgaptmp_001534	hypothetical protein
	**4**	3.11×10^{-38}	3.94	pgaptmp_002986	hasin family protein
	**5**	2.02×10^{-30}	3.34	pgaptmp_000388	superoxide dismutase [Fe]
Glucose	**1334**	8.50×10^{-4}	0.93	pgaptmp_001731	carbohydate-binding domain-containing protein (GH20)
	**1353**	9.96×10^{-4}	0.94	pgaptmp_001323	beta-N-acetylhexosaminidase (GH3)

**1512**	2.89×10^{-3}	0.79	pgaptmp_001504	glycosyl hydrolase family 18 protein
**1900**	2.47×10^{-2}	0.60	pgaptmp_000440	sugar ABC transporter permease
**3390**	9.32×10^{-1}	0.03	pgaptmp_000441	sugar ABC transporter substrate-binding protein

Table S4: Differentially upregulated *J. wiesaeckerbachi* transcripts involved in gene regulation under minimal colloidal chitin growth conditions compared to minimal glucose media derived samples, from highest to lowest log2 fold changes. Chitin-fed *J. wiesaeckerbachi* cells were harvested and RNA subsequently extracted after three days of cultivation, while glucose-induced RNA was extracted after one day of cultivation, in duplicates each.

Gene ID	log2 Fold Change	Adjusted *p*-value	Annotation (PGAP)
pgaptmp_002681	2.53	6.24×10^{-22}	sigma-70 family RNA polymerase sigma factor
pgaptmp_002797	2.41	1.12×10^{-9}	Rrf2 family transcriptional regulator
pgaptmp_000237	2.21	2.66×10^{-12}	hypothetical protein (putative TF according to SWISS-MODEL)
pgaptmp_000300	2.12	1.88×10^{-13}	anti-sigma factor
pgaptmp_002638	2.12	3.45×10^{-5}	GntR family transcriptional regulator
pgaptmp_000299	2.01	3.29×10^{-12}	sigma-70 family RNA polymerase sigma factor
pgaptmp_001414	1.75	3.74×10^{-7}	LytTR family DNA-binding domain-containing protein
pgaptmp_002188	1.65	1.63×10^{-10}	DeoR/GlpR family DNA-binding transcription regulator
pgaptmp_002870	1.64	2.44×10^{-10}	proline dehydrogenase family protein
pgaptmp_002388	1.64	4.80×10^{-9}	elongation factor G
pgaptmp_001086	1.58	5.97×10^{-8}	methylated-DNA--[protein]-cysteine S-methyltransferase
pgaptmp_000877	1.54	4.80×10^{-9}	sigma-54 dependent transcriptional regulator
pgaptmp_000768	1.41	6.30×10^{-8}	sigma-70 family RNA polymerase sigma factor
pgaptmp_002499	1.41	4.72×10^{-6}	sensor histidine kinase KdpD
pgaptmp_000768	1.35	2.87×10^{-7}	trifunctional transcriptional regulator/proline dehydrogenase/L-glutamate gamma-semialdehyde dehydrogenase
pgaptmp_000895	1.23	2.05×10^{-6}	sigma-54 dependent transcriptional regulator
pgaptmp_002703	1.22	1.14×10^{-3}	metalloregulator ArsR/SmtB family transcription factor
pgaptmp_003366	1.21	3.92×10^{-2}	GntR family transcriptional regulator

Table S5: Bioinformatic transcriptomics analysis parameters as performed and provided by Eurofins Genomics.

Step	Tool	Parameter
Alignment	STAR run through Sentieon framework	–twopassMode Basic –alignIntronMax 1–limitBAMsortRAM 300000000000 –outFilterMultimapNmax 20 –outMultimapperOrder Random –outReadsUnmapped None –outSAMmapqUnique 255 –outSAMattributes NH HI AS NM MD XS
Variant Annotation	GATK	-U ALLOW_SEQ_DICT_INCOMPATIBILITY -G StandardAnnotation -G RankSumTest -G WorkInProgressAnnotation -XA MVLikelihoodRatio -A BaseCounts -A AlleleBalance-BySample -A MappingQualityZeroBySample -dt NONE
Gene pred.		default
rRNA purging	RiboDetector	-e rrna –chunk_size 5000
Strand Specificity	Rseqc	default
Sequence Cleaning	Seqtk	–length_required 80 –qualified_quality_phred 20 –average_qual 20 –detect_adapter_for_pe
	snpEff	-noLog -no-downstream -no-upstream -no-intergenic
Split Read Junction	Sentieon	–reassign_mapq 255:60
Transcript Quantification	featureCounts	–donotsort -p -C –largestOverlap -g ID -t gene -s 2 -T 8 -Q 10
Variant Calling	Sentieon's HaplotypeCaller	–call_conf 20 –emit_conf 20 –trim_soft_clip
Variant Filtration Indel	GATK's Variant Filtration module	–filterExpression 'QD < 2.0' –filterName QDFilter –filterExpression 'ReadPos-RankSum < -20.0' –filterName ReadPosFilter –filterExpression 'FS > 200.0' –filterName FSFilter
Variant Filtration	GATK's Variant Filtration module	–filterExpression 'DP <= 20' –filterName LowCovFilter –filterExpression 'QD< 2.0' –filterName QDFilter –filterExpression 'MQ < 40.0' –filterName MQFilter –filterExpression 'FS> 60.0' –filterName FSFilter –filterExpression 'HaplotypeScore > 13.0' –filterName HaplotypeFilter –filterExpression 'MQRankSum < -12.5' –filterName MQRankSumLow –filterExpression 'ReadPos-RankSum < -8.0' –filterName ReadPosFilter

Table S6: CAZyme prediction of the collective >1.2-fold upregulated proteomic and transcriptomic results of *J. wiesaeckerbachi* with chitin substrate, mediated by dbCAN 3.0. AA = auxiliary activity, CBM = carbohydrate binding module, CE = carbohydrate esterase, GH = glycosyl hydrolase family, GT = glycosyltransferase. The two sequence homology based function prediction tools HMMER and DIAMOND are deployed by dbCAN3.0 with the following parameters: HMMER:dbCAN (E-Value < 10^{-15}, coverage > 0.35), DIAMOND: CAZy (E-Value < 10^{-102}) HMMER: dbCAN-sub (E-Value < 10^{-15}, coverage > 0.35). SignalP predicts prokaryotic signal peptides of the conventional Sec, TAT and pilin-like translocons, processed with either of SPI, SPII or SPIII leader peptidases, respectively.

Gene ID	HMMER	dbCAN_sub	DIAMOND	SignalP
pgaptmp_000148	AA10(23-202)	AA10_e25+CBM5_e79	AA10+CBM5	Y(1-23)
pgaptmp_000203	N	N	CBM12+CBM5	Y(1-21)
pgaptmp_000269	GH20(299-720)	GH20_e124	GH20	N
pgaptmp_000302	GH19(265-520)	GH19_e2	CBM5+GH19	Y(1-22)
pgaptmp_000306	GH20(306-724)	GH20_e124	GH20	N
pgaptmp_000366	CBM5(125-168)+GH18(247-668)	CBM12_e3+CBM5_e51+GH18_e258	CBM12+CBM5+GH18	Y(1-24)
pgaptmp_000371	GH18(344-779)	CBM12_e3+GH18_e326	CBM12+CBM5+GH18	N
pgaptmp_000372	CBM5(114-156)+GH18(218-489)	CBM5_e95+GH18_e14	CBM12+CBM5+GH18	Y(1-25)
pgaptmp_000389	GH18(245-670)	CBM12_e3+CBM5_e51+GH18_e258	CBM12+CBM5+GH18	Y(1-24)
pgaptmp_000437	CE4(62-182)	CE4_e257	CE4	Y(1-21)
pgaptmp_000464	N	N	CBM50+GH25	N
pgaptmp_000470	CE9(13-380)	CE9_e34	CE9	N
pgaptmp_000635	GH18(9-348)	GH18_e233	GH18	N
pgaptmp_000680	GH19(124-224)	GH19_e86	GH19	N
pgaptmp_000744	N	N	GH13	N
pgaptmp_000784	GT4(192-329)	GT4_e4016	GT4	N
pgaptmp_000836	CBM5(115-157)+GH18(243-540)	CBM12_e3+CBM5_e51+GH18_e151	CBM12+CBM5+GH18	Y(1-22)
pgaptmp_000837	GH18(253-674)	CBM12_e3+CBM5_e51+GH18_e164	CBM12+CBM5+GH18	Y(1-24)
pgaptmp_000915	GT51(59-232)	GT51_e153	GT51	N
pgaptmp_000961	N	N	CBM32	Y(1-32)
pgaptmp_001130	GH166(30-250)	GH166_e1	GH166	N
pgaptmp_001135	GT4(315-471)	GT4_e324	GT4	N
pgaptmp_001157	N	AA2_e1	AA0	N
pgaptmp_001207	N	N	GH6	N
pgaptmp_001221	N	N	GT58	N
pgaptmp_001255	N	CBM12_e3	N	Y(1-28)
pgaptmp_001300	GT2(85-251)	GT2	GT2	N
pgaptmp_001323	GH3(64-291)	GH3_e28	GH3	N
pgaptmp_001349	GT28(187-345)	GT28_e46	GT28	N
pgaptmp_001374	N	GH16_e73	GH16	Y(1-24)
pgaptmp_001504	GH18(39-388)	GH18_e153	GH18	Y(1-23)
pgaptmp_001722	GH23(474-603)	GH23_e952	GH23	Y(1-19)
pgaptmp_001732	GH18(245-703)	GH18_e224	CBM12+GH18	Y(1-51)

pgaptmp_001746	GH18(21-488)	GH18_e224	GH18	N
pgaptmp_001840	N	N	CBM12	Y(1-21)
pgaptmp_001841	GH18(21-372)	GH18_e425	GH18	Y(1-19)
pgaptmp_001842	N	N	GH0	N
pgaptmp_001849	GH109(5-188)	N	N	N
pgaptmp_001854	N	CBM5_e49	CBM5	Y(1-24)
pgaptmp_001856	N	N	GT51	N
pgaptmp_001891	N	N	GT2	N
pgaptmp_001955	N	N	GH1	N
pgaptmp_001958	N	N	GH38	N
pgaptmp_002116	GT4(176-299)	GT4_e3056	GT4	N
pgaptmp_002133	CE1(152-377)	N	CBM5	Y(1-27)
pgaptmp_002137	GH18(28-385)	GH18_e316	GH18	Y(1-18)
pgaptmp_002139	N	N	GH1	N
pgaptmp_002189	N	N	GH36	N
pgaptmp_002212	CE2(195-406)	CBM5_e51+CE2_e8	CBM5+CE2	Y(1-21)
pgaptmp_002223	N	N	GT83	N
pgaptmp_002276	N	N	GH28	N
pgaptmp_002558	GH104(11-156)	GH104_e0	N	N
pgaptmp_002718	N	N	GT1	N
pgaptmp_002719	N	N	GT1	N
pgaptmp_002720	N	N	GT1	N
pgaptmp_002942	GH5_28(25-366)	GH5_e210	GH5_28	N
pgaptmp_002985	N	N	GH13	N
pgaptmp_002996	AA7(31-458)	AA7_e0	N	N
pgaptmp_003049	GH9(102-537)	GH9_e20	GH9	N
pgaptmp_003078	GH103(32-321)	GH103_e29	GH103	Y(1-21)
pgaptmp_003083	GH18(198-582)	GH18_e427	GH18	Y(1-18)
pgaptmp_003133	N	CBM50_e508	CBM50	Y(1-26)
pgaptmp_003160	N	N	GH13_31	N
pgaptmp_003182	N	N	GT2	N
pgaptmp_003195	N	N	CE12	N
pgaptmp_003266	GH23(302-444)	GH23_e322	GH23	N
pgaptmp_003309	GH23(334-469)	GH23_e69	GH23	Y(1-32)
pgaptmp_003327	GH102(90-247)	GH102_e23	GH102	N
pgaptmp_003356	N	N	GT2	N
pgaptmp_003359	N	N	GT2	N
pgaptmp_003385	N	N	GH13_11	N
pgaptmp_003521	N	N	CBM50	Y(1-24)
pgaptmp_003548	N	N	GT4	N
pgaptmp_003567	N	CBM50_e665	N	N
pgaptmp_003695	GT4(315-471)	GT4_e324	GT4	N

Full length protocol for in-gel tryptic digestion (timsTOF LC/MS-MS sample preparation)

Day 1:

- Excise gel bands, cut into 1 mm^3 cubes and put into a 1,5 ml sterile micro reaction tube (gel pieces can be stored at -20°C)

General notes:

- The following steps are all done with an Eppendorf ThermoMixer.
- Please note that keratin pollution must be minimised (lab coat, gloves, bouffant cap, hygiene mask).
- All solutions must be stored in glass bottles (to reduce contamination with organic stuff from plastic) and only be refilled with substance (no dish washer, because of detergents).

Solvents:

50 mM ABC (0.2 g ammoniumbicarbonate in 50 ml H_2O_{dd} (HPLC grade)

1:1 ACN:ABC (50 mM) (1:1 mixture of acetonitrile and ABC-buffer)

10 mM DTT (3.1 mg dithiothreitol in 2 ml 50 mM ABC)

55 mM IAA (20.3 mg iodoacetamide in 2 ml 50 mM ABC)

5% FA (5 ml formic acid in 95 ml H_2O_{dd} (HPLC grade))

1% FA (diluted 5% FA solution)

25 mM ABC (diluted 50 mM ABC solution)

1) **Washing procedure:**

- Add 100 µl H_2O_{dd}, 15 min, 550 rpm in ThermoMixer
- Remove supernatant
- Add 200 µl 1:1 ACN:ABC (50 mM), 15 min, 550 rpm
- Remove supernatant
- Add 100 µl ACN, 10 min, 550 rpm (shrinking of gel pieces)
- Remove supernatant
- Add 100 µl 50 mM ABC, 5 min, 550 min
- Add additional 100 µl ACN, 15 min, 550 rpm
- Remove supernatant
- Add 100 µl ACN, 10 min, 550 rpm
- In the meantime, boot the speed vac to precool the vapour condenser
- Remove supernatant
- Dry gel pieces in speedvac, 20 min, 0 mbar, RT. Gel pieces should not be blue anymore.
- Set ThermoMixer to 56°C

2) **Reduction, alkylation, washing, digestion:**

- Add 100 µl 10 mM DTT, 45 min, 56 °C, 550 rpm. Set temperature to 22 °C/room temperature afterwards.
- Remove supernatant
- Add 100 µl ACN

- Remove supernatant
- Add 100 µl 55 mM IAA, 30 min in the dark (place aluminium foil over shaker lid), RT, 550 rpm
- Remove supernatant
- Add 100 µl ACN:ABC (50 mM), 15 min, 550 rpm
- Remove supernatant
- Repeat this step 2 times, 3 x in total
- Add 100 µl ACN, 10 min, 550 rpm, precool/boot speed vac
- Remove supernatant
- Dry gel pieces in speedvac, 15 min, 0 mbar, RT
- Add 100 µl digest solution (1 µl trypsin solution (-80 °C freezer, I4, thaw on ice) in 100 µl **25 mM** ABC 5 °C), incubate in the fridge for 10 min
- 37 °C, **300 rpm** over night
- (After one hour) make sure that all gel pieces are covered with at least 1 mm digest solution, if not add more 25 mM ABC

Day 2:

3) extraction of peptides

- Transfer supernatant into a new micro reaction tube. Always collect all supernatants of the following steps in there (1 micro reaction tube per sample)
- Add 100 µl 25 mM ABC to gel pieces, 15 min sonification
- Add additional 100 µl ACN, 15 min
- Transfer supernatant into micro reaction tube
- Add 100 µl 5% FA, 15 min sonification
- Add additional 100 µl ACN, 15 min sonification
- Transfer supernatant into micro reaction tube
- Add 100 µl ACN, 15 min sonification
- Transfer supernatant to micro reaction tube, store gel pieces at -20 °C
- Remove all solvent in speed vac (the time depends on the number of samples, generally about 2.5 h), Peptides can be stored at -80°C

Day of measurement:

4) Preparing Peptides for mass spectrometry

- Dissolve Peptides in 20 µl 1% FA, 10 min sonification
- Preequilibrate centrifugal filters with 20 µl 1% FA (13,3 k, 1 min)
- Discard equilibration solution
- Add Peptide solution to equilibrated filters (13,3 k, 1 min)
- Transfer filtrate to vial
- **Follow the manual of the centrifugal filters for the washing steps**
